# The costs and benefits of scaling up interventions to prevent poor birth outcomes in low-income and middle-income countries: a modelling study

**DOI:** 10.1016/S2214-109X(24)00238-9

**Published:** 2024-08-14

**Authors:** Neff Walker, Austin Heuer, Rachel Sanders, Hannah Tong

**Affiliations:** a Department of International Health, Bloomberg School of Public Health, Johns Hopkins University, Baltimore, MD, USA; b Avenir Health, Glastonbury, CT, USA

## Abstract

**Background:**

We estimated the benefits and costs of a set of preventive interventions that could be delivered during antenatal care to prevent poor birth outcomes, including small-for-gestational-age and preterm births. We built on the assumptions and analyses underlying the *Lancet* Series on small vulnerable newborns (SVNs) and extended that work by incorporating more recent data, focusing only on the subset of preventive interventions, and examining a broader range of effects. A primary aim of the study was to provide a framework that decision makers could use to design programmes for women and children.

**Methods:**

The analyses used the Lives Saved Tool (LiST) to estimate the effects and costs of scaling up the 11 preventive interventions identified in the SVN Series to improve birth outcomes. We used LiST estimates of effects and costs to estimate benefit–cost ratios (BCRs) for two intervention packages (one with interventions proven to improve birth outcomes and one with proven interventions plus interventions with potential to improve birth outcomes) and for the individual interventions in these packages for 80 low-income and middle-income countries (LMICs).

**Findings:**

Both packages of interventions had BCRs more than 1, with a proven package BCR of 7·3 (IQR 5·3–9·1) and a proven plus potential package BCR of 5·8 (4·4–6·9). We found that in all cases the individual interventions had BCRs more than 1, there was a wide range of BCR values for the different interventions, and the BCR varied depending on package and country.

**Interpretation:**

The analyses presented in this Article provide evidence that there are preventive interventions that, if scaled up in LMICs, could have a large effect on child health and provide benefits that greatly exceed the costs.

**Funding:**

Global Affairs Canada.

## Introduction

Mortality in children younger than 5 years has declined rapidly in low-income and middle-income countries (LMICs) in the past two decades.^1^ Most of this decline was driven by interventions that act on causes of death among children older than 1 month. Neonatal mortality (ie, deaths that occur in the first 28 days of life) accounts for over half of mortality in children younger than 5 years in most LMICs. Prematurity is now the largest cause of death in children younger than 5 years.^2^

A 2023 Series published in *The Lancet* focused on small vulnerable newborns (SVNs). SVNs are defined as births in which the child is either premature (ie, gestation <37 weeks), born small for gestational age (SGA), or both.^3^ In that Series, the authors used the Lives Saved Tool (LiST) to estimate that—if delivered at scale (defined as 90% coverage of people who need them) in 81 LMICs—a set of eight preventive interventions and two treatment interventions with proven efficacy could avert 476 000 neonatal deaths (range 181 000–676 000) per year.^3^ The authors reported that scaling up three additional preventive interventions with some evidence of potential effectiveness could avert additional neonatal deaths.

In this Article we present benefit–cost ratios (BCRs) for scaling up two packages of preventive interventions based on the SVN Series in 80 LMICs: (1) the eight prevention interventions with proven efficacy and (2) the eight interventions with proven efficacy plus the three interventions with potential efficacy. We also present BCRs for the individual interventions contained in each package, highlighting variations by country and package.

## Methods

### Study design

This Article presents modelling of costs and benefits of scaling up 11 interventions during pregnancy to prevent poor birth outcomes (table 1). Eight interventions were classified as proven in the SVN series, and three were considered promising in terms of their potential impact based on meta-analyses that found a reduction in poor birth outcomes. These meta-analyses either had data only from high-income settings or included a limited number of studies, or both.^3^ The modelling uses publicly available data at the national level and therefore no ethical clearance or patient consent were sought.

**Table 1:** Interventions, their efficacy in preventing poor birth outcomes, affected fractions, and adjusted RRs used in the scenarios

	**Preterm births**			**Small-for-gestational-age births**			**Stillbirths**		
	Efficacy (95% CI)	Affected fraction	Adjusted RR	Efficacy (95% CI)	Affected fraction	Adjusted RR	Efficacy (95% CI)	Affected fraction	Adjusted RR
**Interventions with proven effectiveness**									
Prevention of malaria in pregnancy*	0·26 (−0·31 to 0·58)	Percentage of women whose first and second births are exposed to falciparum	1·45	0	0	0	0·32 (0·02 to 0·52)	0	0
Balanced energy and protein supplementation	0	Percentage of pregnant women who are food insecure	1	0·29 (0·06 to 0·46)	Percentage of pregnant women who are food insecure	1·41	0·61 (0·2 to 0·81)	Percentage of pregnant women who are food insecure	2·56
Multiple micronutrient supplementation in pregnancy	0·05 (−0·01 to 0·10)	All pregnant women	1	0·08 (0·02 to 0·12)	All pregnant women	1	0·09 (0·02 to 0·15)	All pregnant women	1
Detection and timely treatment of syphilis	0·51 (0·38 to 0·58)	Percentage of pregnant women with syphilis	2·8	0	0	0	0·82 (0·67 to 0·90)	Percentage of stillbirths due to syphilis	1
Screening for asymptomatic bacteriuria with 7-day antibiotic treatment if detected	0·43 (−0·56 to 0·79)	All women tested and if infected treated	1·77	0	0	0	0	0	0
Low-dose aspirin	0·11 (0·02 to 0·19)	Primiparous births plus all later births to women with hypertension	1·1	0	0	0	0	0	0
Progesterone for at-risk births	0·08 (0·00 to 0·16)	All women who had a previous preterm birth	2·28	0	0	0	0	0	0
Psychosocial interventions for smokers	0·07 (−0·11 to 0·23)	Pregnant women who smoke	1·22	0	0	0	0	0	0
**Interventions with potential effectiveness**									
Calcium supplementation	0·19 (−0·02 to 0·36)	Percentage of pregnant women who are calcium deficient	1·23	0	0	0	0	0	0
Zinc supplementation	0·13 (−0·03 to 0·26)	Pregnant women who are zinc deficient	1·12	0	0	0	0	0	0
Omega-3 fatty acid supplements	0·1 (−0·01 to 0·20)	All pregnant women	1	0	0	0	0	0	0

RR=risk ratio.

* Intermittent preventive treatment of malaria for pregnant women.

**Research in context**

**Evidence before this study**

Prematurity is the largest cause of death among children younger than 5 years in low-income and middle-income countries (LMICs). There has been little progress in reducing deaths related to prematurity, mainly due to insufficiencies in delivering interventions proven to prevent poor pregnancy outcomes (eg, stillbirths, premature birth, and small-for-gestational-age birth) to the people who need them. A 2023 Series published in *The Lancet* on small vulnerable newborns proposed a set of interventions that, if implemented, could greatly reduce poor birth outcomes and the resulting neonatal mortality. The Series identified eight preventive interventions with proven efficacy in reducing births that are small for gestational age, premature births, and stillbirths. The efficacy of all these interventions were based on meta-analyses that found significant effects in low-income and middle-income settings. There were also three additional preventive interventions that had some evidence of efficacy for reducing poor birth outcomes. Most of these interventions have not been introduced in most LMICs and for those that have been introduced, coverage remains low in most settings. This inability to attain population coverage is especially noticeable because these interventions could be delivered via existing antenatal care services, which have high rates of utilisation in these countries.

**Added value of this study**

This study estimated the costs, benefits, and potential effects of scaling up delivery of interventions during pregnancy to prevent poor birth outcomes. These analyses found that the interventions of proven efficacy to prevent poor birth outcomes yielded high benefits at low cost, with benefit–cost ratios above 1. This study also produced a framework and tools that countries could use to guide country-specific programme planning.

**Implications of all the available evidence**

These estimates, along with existing data on current antenatal attendance by pregnant women, suggest that the interventions with proven effectiveness addressed in this study could be scaled up in most LMICs, and efforts to confirm the effectiveness of these promising interventions in diverse settings should be prioritised.

### Methods for estimating impact

LiST is a widely used model that estimates the impact of increasing population coverage of interventions on health outcomes, including age-specific and cause-specific mortality and risk factors for morbidity and mortality such as stunting, wasting, and birth outcomes. LiST incorporates the best-available information on intervention efficacy, risk factors, and current intervention coverage at national and often subnational levels. LiST also estimates the direct and incremental costs of scaling up intervention coverage, including options for incorporating markers of service quality. Numerous comparisons between LiST estimates of changes in mortality based on changes in intervention coverage and measured changes in mortality indicate reasonable levels of agreement.^4^, ^5^, ^6^

LiST was modified to allow it to more accurately estimate interventions that reduce the rate of preterm and SGA births using adjusted relative risk for poor birth outcomes for important risk factors. This was done by using estimates of adjusted relative risk for preterm and SGA births drawn from 2022 publications that estimated country-specific estimates of these risks.^7^, ^8^

LiST calculates impact incrementally, calculating the impact of the first intervention on the total number of birth outcomes on which it has an effect, then the impact of the second intervention on the residual number of birth outcomes, and so on, continuing until the impact of all interventions has been calculated. The total reduction in poor birth outcomes due to prevention is not affected by the ordering of the interventions, but attribution of overall effects is. To attribute impact to individual interventions, LiST estimates the total preterm and SGA births averted by each prevention intervention, and then attributes this total to the percentage contribution of each intervention to impact based on its efficacy multiplied by the percentage of the population (affected fraction) and coverage change.

We ran two modelling scenarios (proven and proven plus potential packages of interventions) for each of the 80 LMIC countries that together comprise the overwhelming global burden in maternal, neonatal, and younger than 5 years mortality.

The list of countries is available in the appendix (pp 8–9). For both scenarios, we increased coverage of interventions from the country-specific rate in 2023 to 90% in 2024 and continued 90% coverage up to 2030. We selected 2030 as an endpoint to capture intervention impact on stunting over a 5-year period for the birth cohort. We report results for the year 2030. These scale-up scenarios used the 2030 country-specific model as the counterfactual to estimate deaths averted, changes in mortality numbers and rate, changes in numbers of SGA and preterm births, and changes in rates of stunting and stillbirths and costs. In addition, we ran projections for Bangladesh and Nigeria in which we scaled up each of the 11 interventions individually, allowing us to estimate intervention-specific BCRs independent of the effects of the other interventions.

Changes in birth outcomes also have downstream effects that are captured in LiST. For example, children who are born SGA or preterm are at increased risk of death during the neonatal period. In addition, these children are at greater risk of being stunted, which carries an additional risk of mortality. More details on how LiST handles these relationships between birth outcomes, later growth, and mortality have been published elsewhere.^9^, ^10^, ^11^

### Methods for estimating costs

The LiST costing module estimates the total costs of implementing each intervention on the basis of three components: (1) intervention-specific costs; (2) programme costs (the costs of running the portion of a reproductive, maternal, newborn, and child health programme associated with the intervention); and (3) the broader health system costs associated with providing the intervention from a provider perspective (figure). The cost estimates do not reflect costs to clients, such as transportation or missed wages. The total incremental costs were calculated as the difference of the total costs with and without scale-up of interventions.

**Figure:**
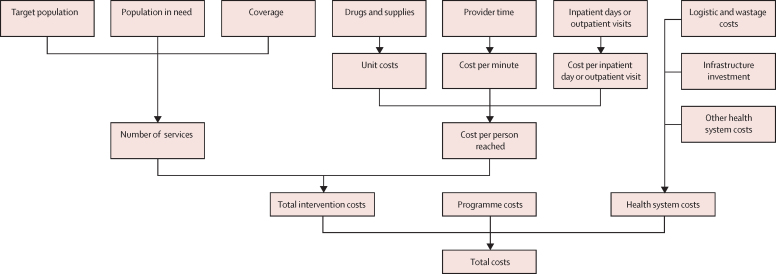
Lives Saved Tool costing module Target population is defined as the population that could possibly receive the intervention. Population in need is used to identify what share of the target population requires the intervention; the proportion is established by incidence, prevalence of condition, and treatment guidelines.

LiST uses an ingredient-based and population-based approach to estimate intervention-specific costs (figure).^12^ Country-specific estimates of the target population, the population in need, and intervention coverage are multiplied to provide an estimate of the required number of services for each intervention per year. The estimated country-specific cost of service delivery per person reached for each intervention is an aggregate of drug and supply prices, the cost of provider time, and the cost per inpatient day or outpatient visit. Further details for intervention-specific costs are available in the appendix (pp 4–6).

Programme costs include costs for communication, training, supervision, monitoring and evaluation, advocacy, and other programme management. The default estimates of the programme costs, drawn from work completed by Results for Development, were 15% of the intervention-specific costs,^13^ applied equally to all interventions. Detailed default programme costs categories and percentages, and guidance on adapting these costs to local circumstances, are available in the appendix (p 7).

Health system costs include logistics and wastage, infrastructure investment, and other health system costs. Logistics costs reflected estimated resource requirements for expanding the supply chain, calculated as 16% of the drug and supply costs. Wastage costs were estimated to be 5% of drug and supply costs. Infrastructure investment assumes that the facility network will need to expand to meet expanding numbers of services required to scale up a given intervention. All the interventions in the analysis were assumed to be add-ons to the existing antenatal care visit so no infrastructure costs were needed. Other health-system costs such as governance and health information systems were estimated based on country income level and applied to intervention-specific costs. The ratios for other health system costs applied for low-income, lower-middle-income, and upper-middle-income countries were 85·6%, 69·9%, and 50·0%, respectively.^14^ Details on our assumptions are available in the appendix (p 2).

### Methods for estimating benefits

Benefits of the intervention packages and individual interventions were considered across three domains: (1) economic benefits due to reductions in stunting; (2) economic benefits from increased workforce participation; and (3) social benefits of saved lives. The calculations for the latter two domains were adapted from the Global Investment Framework.^14^

We estimated the economic benefits due to increased earnings because of reduced stunting in three steps. First, we used LiST stunting outputs to estimate improvements in linear growth. We quantified educational gain using a global estimate of 0·47 additional years of schooling obtained per unit increase in height-for-age Z score among birth cohorts of children who are stunted aged 12–23 months.^15^ Second, we quantified the relative gains in wages, using country-specific estimates of the percentage of wage increase per additional year of schooling obtained based on the relationship between years of schooling and lifetime earnings.^16^ Third, we used country-level data on wages and discount rates to estimate the net present value of future earnings gained due to early childhood growth improvement per birth cohort. We estimated future wages using country-level gross national income (GNI) per capita, a global estimate of labour share of income (50%), and the country-specific labour force participation rate.^17^ We projected GNI per capita in future for 60 years using the estimated country-specific annual growth rate. We held labour share of income and labour force participation rate over time. Because the additional wage earnings will accrue only once for each child as the birth cohort enters the labour market, we used 44 years as the default number of working years, starting at age 16 years. The future annual wages were discounted at 3% to present value in 2030 because the additional lifetime earnings will be gained in the future. The costs are in present value (ie, money to spend in 2030). To make the comparison to calculate BCRs, we need to discount the future gains (both social and economic benefits) to the present value. The discounting calculations are the same for social and economic benefit. A more detailed description of the methods and assumptions is available elsewhere.^18^

We also estimated the economic benefits from increased workforce participation due to the number of lives saved and life-years gained by averting stillbirths and neonatal deaths. One neonatal life saved counted as one life saved. Half of the stillbirths averted were included, reflecting the proportion assumed to happen peripartum.^16^ For this 2030 birth cohort, each person was assumed to be working for 44 years, starting at age 16 years. The yearly wage was estimated to be equivalent to gross domestic product per capita^19^ with a 2% annual growth rate. The latest country-level workforce participation rates (percentage of total population aged 15–64 years) were from the World Development Indicators.^19^ We calculated total economic benefits as the number of statistical lives gained multiplied by 44 years of salary and then multiplied by the workforce participation rate.^19^ We did not project future workforce participation rates. The number of statistical lives gained equalled the number of neonatal lives saved plus 0·5 times the number of stillbirths averted. Future annual wages were discounted at 3% to the value in 2030. The value of 44 years of salary for the 2030 birth cohort was calculated as

$$\sum_{i=0}^{44}=\left(\text{projected GDP per capita in}\;2046\times 1\cdot 02^{i}\right)/\left(1\cdot 03^{i}\right)$$

where *i* represents the given year of working, 2046 represents when the 2030 birth cohort join the workforce, and 44 is the number of working years.

We developed a single estimate across the 80 countries of the social benefits from lower rates of stillbirths and neonatal mortality, leading to fewer lost years of healthy life, because an individual's social value should not be affected by the income status of their country of residence. We calculated a weighted GDP per capita based on the 80 country-level estimates of GDP per capita and population size. The weighted GDP was also estimated to increase 2% annually. Following the procedure by Stenberg and colleagues,^14^ we assumed the social benefits per capita were half the weighted GDP per capita. The social benefits gained per life were estimated based on annual social benefits per capita and the country-specific healthy life expectancy at birth.^20^ A 3% discount rate was applied to future social benefits per capita to estimate the present value of social benefit in 2030. Total social benefits were calculated, where the present 2030 value of social benefits gained per life equalled

$$\sum_{i=0}^{A}=\left(\text{projected GDP per capita in}\;2030*1\cdot 02^{i*}0\cdot 05\right)/\left(1\cdot 03^{i}\right)$$

where *A* is the healthy life expectancy at birth and *i* represents the given year of working. Total social benefits equalled the present value of social benefits gained per life multiplied by the number of statistical lives gained.

The total benefits were calculated as the sum of three domains of economic benefits due to reduced stunting, economic benefits from increased workforce participation, and social benefits of saved lives. Social benefits were calculated in USD as described in the equation, social benefits per capita was quantified to be equivalent to half of weighted GDP per capita, which is in USD. A BCR value more than 1 indicates that the benefits of the intervention are greater than the costs. For BCR we used IQRs from the country-level BCR ranked at the 25th and 75th percentile among the 80 countries.

### Role of the funding source

The funders had no role in the writing of the manuscript or the decision to submit it for publication.

## Results

Table 2 shows the total benefits and total incremental costs for the proven and the proven plus potential intervention packages for a single year of providing 90% coverage for all package interventions. The proven package would cost about US$10·4 billion, and the proven plus potential package would cost about $17·5 billion. These costs are incremental and do not include the costs of maintaining current levels of coverage for these interventions. Table 2 also shows the costs broken down into intervention-specific costs ($4·9 billion for the proven package and $8·4 billion for the proven plus potential package) and the broader health system costs ($5·4 billion and $9·1 billion, respectively) for scaling up these interventions. Table 2 also shows the total and subcategory benefits of each package, with an estimated $74·7 billion benefits for the proven package and $101·4 billion for the proven plus potential package.

**Table 2:** Total costs and benefits of intervention scale-up in 2030

		**Package of interventions with proven effectiveness**	**Package of interventions with proven plus potential effectiveness**
Total incremental costs (US$)		10 339 695 428	17 489 138 162
	Intervention-specific costs	4 928 183 909·18	8 385 568 701
	Programme and health system costs	5 411 511 518·70	9 118 456 370
Total benefits (US$)		74 748 282 831	101 445 105 384
	Additional lifetime earnings due to reduction in stunting	4 820 373 149	6 994 684 450
Economic benefits with increased workforce participation		23 944 108 732	32 712 343 768
Social benefits		45 983 800 950	61 738 077 166

Benefits were discounted at 3% to the present value in 2030. Intervention-specific costs included drug and supply costs and labour costs. Programme and health system costs included programme costs (15% of the intervention-specific costs), logistics and wastage costs (21% of drug and supply costs), and other health system costs (50–86%) of intervention-specific costs depending on country's income status.

In table 3 we present the costs per health outcome averted (stillbirths, neonatal deaths, and stunting cases) for the two packages and by intervention within each package. The total costs per stillbirth and case of stunting averted are lower in the proven package but the cost per neonatal death averted is lower in the proven plus potential package. There are large differences by intervention in the cost per health outcome averted. For example, detection and treatment for asymptomatic bacteriuria has the lowest costs per outcome averted for neonatal deaths and stunting cases but has no effect on stillbirths. Other interventions affect all three outcomes, but the costs per outcome averted vary depending on the efficacy of the intervention and the importance of the risk being addressed by the intervention. For example, protection of pregnant women from malaria in pregnancy has a relatively low cost per stunting case averted among interventions in both packages (US$770 in the proven package and $871 in the proven plus potential package), but its cost per stillbirth averted is much higher ($6510 in both packages). This variability in cost across health outcomes highlights the need for a composite health outcome measure (eg, BCR) as one aspect of evaluating the possible costs and effects of interventions.

**Table 3:** Cases averted and cost per outcome averted in 2030 by intervention package and intervention, in US$

		**Package of interventions with proven effectiveness**			**Package of interventions with proven and potential effectiveness**		
		Stillbirth	Neonatal death	Stunting	Stillbirth	Neonatal death	Stunting
Total cases averted		819 383	277 507	3 641 107	819 383	513 745	5 193 545
Interventions with proven effectiveness							
	Balanced energy and protein supplements	13 211	264 644	6265	13 211	284 331	6367
	Low-dose aspirin	NA	3524	497	NA	3955	558
	Multiple micronutrient supplements	7968	16 890	1003	7968	18 727	1049
	Prevention of malaria in pregnancy*	6510	3844	770	6510	4354	871
	Progesterone for at-risk births	NA	23 526	3423	NA	26 362	3839
	Psychosocial interventions for smokers	NA	5739	920	NA	6438	1035
	Detection and timely treatment of syphilis	4710	19 403	4465	4710	21 148	5032
	Screening for asymptomatic bacteriuria with 7-day antibiotic treatment if detected	NA	868	127	NA	974	142
Interventions with potential effectiveness							
	Calcium supplements	NA	NA	NA	NA	30 352	4909
	Omega-3 fatty acid supplements	NA	NA	NA	NA	29 970	4375
	Zinc supplements	NA	NA	NA	NA	15 612	2168
Total package		12 619	37 259	2840	21 344	34 043	3367

NA=not applicable because intervention has no shown efficacy against the outcome (table 1) or because the intervention is not included in the package, or both.

* Intermittent preventive treatment of malaria for pregnant women.

We present the results of the BCR analysis in table 4. Both packages and the individual interventions had BCRs more than 1 in all scenarios. The BCR for the proven package was 7·3 (IQR 5·3–9·1) and for the proven plus potential package was 5·8 (4·4–6·9). Columns four and five show the same BCR results for Bangladesh and Nigeria as exemplar countries. Columns six and seven show the BCRs for each of the 11 interventions in Bangladesh and Nigeria in a scenario where only a single intervention was scaled up to 90% coverage, and coverage for the other interventions remained at current levels.

**Table 4:** BCR by intervention package and intervention

		BCR for 80 LMICs in the proven package*	BCR for 80 LMICs in the proven plus potential package*	**BCR for Bangladesh in the proven plus potential package**	**BCR for Nigeria in the proven plus potential package**	**BCR for Bangladesh in individual intervention scale-up**	**BCR for Nigeria in individual intervention scale-up**
Interventions with proven effectiveness							
	Balanced energy and protein dietary supplements	4·3 (3·2–5·3)	4·3 (3·2-5·3)	5·6	5·3	6·0	5·9
	Low-dose aspirin	32·5 (26·4–43·5)	29·0 (23·2–39·3)	19·2	39·3	23·8	49·1
	Multiple micronutrient supplements	14·1 (10·3–16·7)	13·5 (9·7–15·9)	13·5	17·1	15·1	20·2
	Prevention of malaria in pregnancy†	33·2 (30·3–55·9)	30·2 (27·8–50·9)	NA	28·2	NA	34·4
	Progesterone for at-risk births	4·7 (3·7–6·3)	4·2 (3·2–5·6)	2·6	5·7	3·2	7·1
	Psychosocial interventions for smokers	19·2 (14·1–25·3)	17·1 (12·7–21·3)	15·7	NA	19·3	NA
	Detection and timely treatment of syphilis	15·7 (8·7–28·0)	15·3 (8·6–27·1)	5·1	27·5	5·6	31·3
	Screening for asymptomatic bacteriuria with 7-day antibiotic treatment if detected	130·6 (102·2–159·5)	116·5 (90·9–142·7)	91·3	119·2	113·3	148·9
Interventions with potential effectiveness							
	Calcium supplements	NA	3·7 (3·0–5·9)	3·4	6·9	4·2	8·6
	Omega-3 fatty acid supplements	NA	3·8 (2·9–5·1)	2·4	5·4	2·9	6·7
	Zinc supplements	NA	7·2 (2·9–9·1)	4·5	10·4	5·6	13·0
Total package		7·3 (5·3–9·1)	5·8 (4·4–6·9)	5·4	7·8	NA	NA

BCR=benefit–cost ratio. LMIC=low-income and middle-income country. NA=not applicable because no malaria in Bangladesh or no pregnant women who were smokers in Nigeria according to the source.

* Data are BCR (IQR), with the lower bound the country-level BCR ranked at the 25th percentile among the 80 countries and the upper bound the country-level BCR ranked at the 75th percentile among the 80 countries.

† Intermittent preventive treatment of malaria for pregnant women.

## Discussion

Our results support previous work showing the positive benefits of implementing packages of interventions to prevent poor birth outcomes for pregnant women in LMICs.^3^ We found, however, that BCRs for individual interventions within a package varied widely. For example, the BCRs for screening and treatment for asymptomatic bacteriuria in the proven and proven plus potential packages were roughly 18 times higher than the BCRs for the two packages. Two other interventions—prevention of malaria in pregnancy and low-dose aspirin for at-risk pregnancies—had BCRs of around 30. On the other end of the spectrum, five (45%) of the 11 interventions in the proven plus potential package had BCRs less than 8.

We also found that the estimates of benefits and costs for an intervention could vary based on the package within which it was embedded. This finding is illustrated by the estimated BCRs for individual interventions in Bangladesh and Nigeria when they were scaled up in isolation, rather than as part of a package. When several interventions within a package affect the same or a related health outcome, the BCRs for the individual interventions will be higher if scaled in isolation. This pattern is because the costs remain constant, but the health impact is no longer shared. These findings suggest that packages of interventions should also look at effects of interventions scaled up individually in specific settings, to prioritise interventions that will have the greatest effect at lowest cost.

There were also important variations in intervention-specific BCRs based on epidemiological and health system contexts, as reflected in the IQRs for the BCRs and the different BCRs for Bangladesh and Nigeria. In general, the BCRs were lower in Bangladesh than in Nigeria (BCRs for the proven plus potential package were 5·4 and 7·8, respectively). This variation could be driven by country-specific differences in the costs of providing services, and by differences in the prevalence of diseases and risk factors. For example, syphilis detection and treatment had a much larger BCR in Nigeria than in Bangladesh, attributable to a higher prevalence of syphilis in pregnant women.^21^ However, providing balanced energy and protein supplements to pregnant women had a larger BCR in Bangladesh (5·6) than in Nigeria (5·3). These findings suggest that aggregated analyses, such as those presented in this Article (eg, interventions grouped in packages and countries grouped by income status or geographical location), are only a starting point for the more decentralised decision making that will generate gains for women and children. The next step is for country decision makers to develop models that incorporate specific needs and implementation plans to develop the most beneficial and feasible scenarios for their settings. To aid in this process, we have made the country models available on the LiST website.

Our analysis has important limitations. First, as with all modelling efforts, our estimates are only as good as our data inputs and the transparency of our assumptions. We have included full details of methods and assumptions in the appendix (p 11), and we welcome replications and refinements. Second, we have shown that individual interventions can have important differences in costs for different outcomes. Although BCRs provide a useful and necessary summary measure across outcomes, country planners will need to review the outcome-specific results when making decisions about their priorities for implementation. Third, the two package scenarios presented in this Article are not meant to represent attainable scale-up functions for all countries, nor do we assume that countries will scale up all or even most of these interventions simultaneously. Rather, these scenarios are aspirational and represent the maximum effect of an ambitious scale-up of these interventions across a broad set of LMICs. Our intention is to show potential costs and benefits, and how these could vary by package and by country. Finally, all three of the potential interventions, and perhaps some of the proven interventions, will need more data on efficacy and effectiveness before countries should commit to bringing these interventions to scale.

Despite these limitations, our findings suggest that many poor birth outcomes can be averted in LMICs through the delivery of interventions via antenatal care visits at reasonable cost. Many previous reproductive, maternal, newborn, and child health benefit–cost analyses have not addressed adequately the scalability of the interventions, and especially the feasibility of attaining the levels of coverage assumed in the modelling scenarios in LMICs. For example, although providing comprehensive emergency maternal and obstetric care (CEMOC) or immediate kangaroo mother care (iKMC) with full supportive care^22^ could save many neonatal lives, scaling up these interventions would be very difficult and costly for most LMICs, requiring a massive expansion of facilities able to provide CEMOC, more trained providers, and increased access to these high-level facilities by pregnant women. Although ensuring services such as CEMOC and iKMC by 2030 is a worthy goal, the results presented here suggest that there are more immediate and affordable options that could be implemented in LMICs.

We know that service provision is not enough. Women must also access and use these services to realise the benefits and save young lives. In most LMICs, high proportions of women are already accessing antenatal care services. For the 80 countries in this analysis, an average of 81·5% of people who gave birth reported at least one antenatal visit, and 53·8% had four or more antenatal visits. Antenatal care contacts are an important missed opportunity for attainable health gains,^23^, ^24^ and our findings offer a way to fill this gap with a limited set of interventions with moderate-to-high BCRs that can prevent poor birth outcomes and reduce stillbirths, neonatal mortality, and stunting among children.

## Contributors

## Data sharing

The country-specific Lives Saved Tool (LiST) models are available for download from the LiST website without restriction.

## Declaration of interests

NW reports a grant from Global Affairs Canada and the Bill & Melinda Gates Foundation. All other authors declare no competing interests.
