# Supplementary material for: The costs and benefits of scaling up interventions to prevent poor birth outcomes in low-income and middle-income countries: a modelling study
Source: Lancet Glob Health. 2024 Aug 14;12(9):e1526–33. doi: 10.1016/S2214-109X(24)00238-9 (PMC11345446; doi:10.1016/S2214-109X(24)00238-9)
Supplement: Supplementary appendix [file mmc1.pdf]

THE LANCET
Global Health

Supplementary appendix

This appendix formed part of the original submission and has been peer reviewed. We post it as supplied by the authors.

Supplement to: Walker N, Heuer A, Sanders R, Tong H. The costs and benefits of scaling up interventions to prevent poor birth outcomes in low-income and middle-income countries: a modelling study. *Lancet Glob Health* 2024; **12:** e1526–33.

Supplemental Material

Details on costing inputs -- 2

Supplemental Table 1: Intervention-specific costing assumptions -- 4

Supplemental Table 2: Program cost categories and values -- 7

Supplemental Table 3: Benefit cost ratio for 80 countries in the proven package and the proven and potential package -- 8

Supplementary table 4: Lives saved and costs by intervention in the proven and the proven and potential package -- 10

Link to LiST models for the 81 countries and spreadsheets for BCR calculations -------------------------------- 11

Details on costing assumption

As described in the main body of the article, service delivery costs are estimated based on volume of clients and include cost categories for drugs and consumables, labor, and the costs of inpatient days and outpatient visits. Costs for drugs and consumables were supplied by the UNICEF supply catalogue[1] and MSH International Drug Price Indicator Guide[2]. Costs for staff salaries prepopulated with assumptions for salaries, benefits, and time utilization were drawn from WHO CHOICE. Costs for inpatient days and outpatient visits were also drawn from WHO CHOICE estimates[3]. See Supplemental Table 1 for detailed intervention-specific costing inputs.

Target populations and those in need of services are estimated based on demographic projections, LiST estimates for incidence and etiology, and literature on incidence and prevalence of various conditions. The cost per case is estimated using an ingredients approach which incorporates quantities and cost of drugs and supplies, provider time, and numbers of inpatient and outpatient visits from the One Health Tool databases developed with WHO[4].

RMNCAH-N program costs such as supervision, training, monitoring and evaluation are calculated as an additional percentage of intervention costs. Default program cost categories and percentages (Supplemental Table 2.) have been provided, based on SUN nutrition plan costing exercises, the EPIC immunization studies, and National AIDS Spending Assessments[5]. Users are encouraged to adapt these cost categories and the assumed level of costs based on recent country-specific data if available. Users have the option of configuring the program costs categories and entering costs either as a percentage of direct costs, or as an absolute number.

Health system costs are captured based on a structure that disaggregates supply chain costs, wastage costs, infrastructure investments, and other health system costs such as governance and health information systems. The estimation of costs for logistics was applied as a mark-up rate to the value of commodity, including dugs and supplies, costs in order to approximate resource requirements for expanding the supply chain. We used the average percentage of commodity value needed for expanding the supply chain in a number of countries with varied baseline logistics system condition[6]. Wastage costs were estimated to be 5% of commodity costs to reflect that certain quantity of drugs were not used past the expiration date.

Infrastructure investment assumes that the facility network will need to expand to meet expanding numbers of services required to scale up a given plan[6]. The investment required is estimated based on the change between each year's number of services and the associated number and costs of inpatient days and outpatient visits and includes facility construction, medical equipment, and furniture costs required to expand the network in order to realistically roll out the service package being analyzed. No infrastructure investment is needed for the analysis because all the interventions in the packages were assumed to be add-ons of the existing antenatal care visits.

Other health system costs such as governance and health information systems are estimated based on country income level and applied to intervention-specific costs. A ratio of other health system costs to intervention-specific costs was derived from previous WHO work[6] that includes a disaggregated analysis of costs by country for service delivery in RMNCH. This has been adapted for LiST Costing to allow estimation of other health system costs for a given package of care or an individual intervention.

Reference:

1. UNICEF Supply Catalogue . [Internet]. UNICEF. Available from: https://supply.unicef.org/
2. MSH (Management Sciences for Health). 2016. International Medical Products Price Guide, 2015 Edition. Medford, Mass.: MSH.
3. Gkountouras, Lauer, Stanciole, Stenberg, and Tan-Torres Edejer. Estimation of unit costs for general health services: Updated WHO-CHOICE estimates. World Health Organization. Geneva, Switzerland.
4. Avenir Health. OneHealth Tool Intervention Assumptions. 2016. Accessible https://avenirhealth.org/Download/Spectrum/Manuals/Treatment%20Assumptions%202016%201%2010.pdf
5. Clift, Aria, Chaitkin, et al. Landscape Study of the Cost, Impact, and Efficiency of Above Service Delivery Activities in HIV and Other Global Health Programs. Results for Development. 2016. Washington, DC.

6. Stenberg, K., H. Axelson, P. Sheehan and others, 2014. “Advancing Social and Economic Development by Investing in Women's and Children's Health: A New Global Investment Framework.” The Lancet 383(9925): 1333-54.

Supplemental Table 1: Intervention-specific costing assumptions

Drugs and supply assumptions

Drug/Supply	Percent receiving this aspect of the treatment	Note	Number of units	Times per day	Days per case	Units per case	Unit cost USD	Cost per average case USD
**Prevention of malaria in pregnancy**								
Sulfamethoxazole + trimethropin, tablet 400 mg + 80 mg	All pregnant women in countries with IPTp policy	Intermittent preventive treatment (IPT) at least 2x during 2nd and 3rd trimester	1	1	6	6	0·01	0·06
Total cost								0·06
**Balanced energy supplementation**								
High energy biscuit, 100 grams	Pregnant women who are food insecure		1	3	180	540	0·12	64·8
Total cost								64·8
**Multiple micronutrient supplementation in pregnancy**								
Micronutrient, film-coated tablet	All pregnant women	100% of pregnant women for 6 months of pregnancy	1	1	180	180	0·02	3·6
Total cost								3·6
**Calcium supplementation**								
Calcium, tablet, 600 mg	All pregnant women	1·5-2 g a day	3	1	180	540	0·02	10·8
Total cost								10·8
**Zinc supplementation in pregnancy**								
Zinc, tablet, 20 mg	All pregnant women		1	1	180	180	0·01	2 ·47
Total cost								2 ·47
**Syphilis detection and treatment**								
Screening	All pregnant women							
Blood collecting tube, 5 ml		For taking blood sample	1	1	1	1	0·24	0·24
Gloves, exam, latex, disposable, pair			1	1	1	1	0·06	0·06
Syringe, needle + swab		For taking blood sample	1	1	1	1	0·05	0·05
Test, Rapid plasma 4egain (RPR)		Syphilis test	1	1	1	1	0·13	0·13
Total costs for screening								0·48
Treatment	Pregnant women who test positive for syphilis	Based on country-specific estimate of syphilis prevalence						
Benzathine benzylpenicillin, powder for injection, 2·4 million IU		Antibiotic	1	1	1	1	0·26	0 ·26
Syringe, needle + swab		for benzathine benzylpenicillin injection	1	1	1	1	0·05	0 ·05
Water for injection, 5 ml ampoule		for benzathine benzylpenicillin injection	1	1	1	1	0·05	0 ·05
Total cost for treatment								0·36
**Screening and Treatment of bacteriuria**								
Test strips, urine analysis	All pregnant women		1	1	1	1	0·047	0·047

Total cost for testing								0·047
Nitrofurantoin, 100mg tab	Women who test positive for ASB	For all countries used 11% as estimate of ASB prevalence in LMIC	1	2	5	10	0·05	0·5
Total cost for treatment								0·5
**Omega 3 supplements**								
Omega 3 fish oil capsule, 1000 mg	All pregnant women	2,000 mg per day	2	1	112	224	0·04	8·60
Total cost								8·60
**Low dose aspirin**								
Acetyl salysilic acid (aspirin), tablet, 75 mg	All pregnancies where there was previous history of pre-eclampsia or preterm birth and all first birth		1	1	112	112	0·01	1·12
Total cost								1·12
**Progesterone**								
Progesterone, 100 mg	At risk pregnant women	All first births plus all later pregnancies where the women had a premature birth (based on country-specific preterm birth rate)	106	1	1	106	0·115	12·19
Total cost								12·19

Provider time assumptions

Staff type	Percent treated by	Note	Minutes	Number of days/visits	Total minutes
**Prevention of malaria in pregnancy**					
Assistant nurses and midwives	100	Added to ANC	2·5	2	5
**Balanced energy supplementation**					
Midwives	100	Added to ANC	2	4	8
**Multiple micronutrient supplementation in pregnancy**					
Midwives	100	Added to ANC	2	4	8
**Calcium supplementation**					
Midwives	100	Added to ANC	2	4	8
**Zinc supplementation in pregnancy**					
Midwives	100	Added to ANC	2	4	8
**Syphilis detection and treatment**					
Midwives	100	Screening	15	1	15
Midwives	20	Injection	5	1	5
Midwives	20	Follow-up	5	1	5
**Treatment of bacteriuria**					
Midwives	100	Added to ANC	3	1	3
**Omega 3 supplements**					
Midwives	100	Added to ANC	2	2	4

**Low dose aspirin**					
Midwives	100	Added to ANC	2	2	4
**Progesterone**					
Midwives	100	Orientation	10	1	10
Midwives	100	Follow up	5	3	15
**Stop smoking education**					
Midwives	100	Counseling provided during ANC visits	10	4	40

Outpatient visits

No outpatient visits for the interventions included in the analysis. All interventions are assumed to be provided as add-ons to existing ANC visits.

Supplemental Table 2: Program cost categories and values

Programme-specific human resources	1%
Training	1%
Supervision	2%
Monitoring and evaluation	2%
Infrastructure	2%
Transport	2%
Communication, media, and outreach	1%
Advocacy	1%
General programme management	2%
Community health worker training	1%
Total	15%

Supplemental Table 3: Benefit cost ratio for 80 countries in the proven package and the proven and potential package·

Country	ISO	Proven package	Proven and potential package
Afghanistan	AFG	4·6	4·2
Algeria	DZA	6·8	4·2
Angola	AGO	6·6	6·2
Azerbaijan	AZE	7·5	3·6
Bangladesh	BGD	7·6	5·4
Benin	BEN	6·2	5·8
Bhutan	BTN	9·4	3·8
Bolivia	BOL	12·8	6·6
Burkina Faso	BFA	6·1	5·3
Burundi	BDI	7·6	7·7
Cambodia	KHM	3·3	2·8
Cameroon	CMR	7·5	6·6
Central African Republic	CAF	5·4	4·9
Chad	TCD	8·7	7·1
Comoros	COM	6·5	6·1
Congo	COG	4·5	4·2
Côte d'Ivoire	CIV	11·5	9·9
Dem· People's Republic of Korea	PRK	1·7	1·5
Democratic Republic of the Congo	COD	6·2	5·6
Djibouti	DJI	10·1	8·9
Dominican Republic	DOM	14·7	14·1
Equatorial Guinea	GNQ	37·9	21·3
Eritrea	ERI	5·4	4·5
Ethiopia	ETH	7·3	6·9
Gabon	GAB	20·8	12·0
Gambia	GMB	5·5	4·8
Ghana	GHA	9·5	7·2
Guatemala	GTM	10·2	8·7
Guinea	GIN	6·8	6·5
Guinea-Bissau	GNB	5·1	4·9
Guyana	GUY	35·4	19·0
Haiti	HTI	4·8	5·0
Honduras	HND	4·4	3·8
India	IND	7·0	4·7
Indonesia	IDN	12·2	5·9
Iraq	IRQ	15·8	7·5
Jamaica	JAM	9·1	6·7
Kenya	KEN	6·5	5·6
Kyrgyzstan	KGZ	5·0	2·0
Lao People's Democratic Republic	LAO	5·2	4·0
Lesotho	LSO	8·1	7·8
Liberia	LBR	5·3	5·2
Madagascar	MDG	4·9	4·5
Malawi	MWI	3·8	3·8
Mali	MLI	7·4	5·7
Mauritania	MRT	7·1	5·2
Morocco	MAR	6·3	4·7
Mozambique	MOZ	4·4	4·4
Myanmar	MMR	5·3	3·8
Namibia	NAM	11·8	10·4
Nepal	NPL	3·3	2·4
Nicaragua	NIC	9·3	3·8
Niger	NER	5·9	4·8
Nigeria	NGA	8·7	7·8
Pakistan	PAK	12·5	8·4
Panama	PAN	34·6	14·8
Papua New Guinea	PNG	8·4	6·4
Paraguay	PRY	9·5	6·3
Philippines	PHL	4·9	4·1
Rwanda	RWA	5·3	4·6
Senegal	SEN	7·5	6·4

Sierra Leone	SLE	4·1	4·1
Solomon Islands	SLB	6·1	4·2
Somalia	SOM	6·6	6·4
South Africa	ZAF	16·4	10·4
South Sudan	SSD	5·6	5·4
Sudan	SDN	7·5	5·6
Suriname	SUR	8·4	6·7
Swaziland	SWZ	7·2	7·4
Tajikistan	TJK	5·8	2·8
Timor-Leste	TLS	7·3	6·1
Togo	TGO	5·1	4·7
Turkmenistan	TKM	10·9	9·2
Uganda	UGA	3·8	3·5
United Republic of Tanzania	TZA	6·6	5·7
Uzbekistan	UZB	3·2	1·9
Venezuela	VEN	29·5	16·7
Yemen	YEM	7·1	4·9
Zambia	ZMB	5·0	5·1
Zimbabwe	ZWE	5·7	5·6

Supplementary table 4: Lives saved and costs by intervention in the proven and the proven and potential package·

	Number of lives saved in the proven package	Number of lives saved in the proven and potential package	Incremental costs (US dollar)
Balanced energy and protein dietary supplements	661 377	659 200	8 321 738 838
Low dose aspirin	57 803	51 516	203 726 666
Multiple micronutrient supplements	209 132	202 558	1 132 203 261
Prevention of malaria in pregnancy*	23 221	21 509	56 115 618
Progesterone	13 780	12 298	324 196 358
Psychosocial interventions for smokers	1 649	1 470	9 464 441
Detection and timely treatment of syphilis	58 845	57 896	223 021 755
Screening for asymptomatic bacteriuria with 7-day antibiotic treatment is detected	79 704	71 071	69 221 574
Calcium supplements	NA	115 487	3 505 222 796
Omega-3 fatty acid supplements	NA	92 079	2 759 623 103
Zinc supplements	NA	56 665	884 647 152

NA – not applicable
*Intermittent preventive treatment of malaria for pregnant women
Total number of lives saved included number of stillbirths prevented and number of neonatal lives saved

Link to LiST models for the 81 countries and spreadsheets for BCR calculations.

The LiST projections files for the 81 countries used in this paper can be downloaded and used with the LiST desktop version of the software or the web-based version, LiST online. To download the countries projections, use the following link: https://www.livessavedtool.org/past-work-by-list-team. Projections with prefix “01LiST1” are for PROVEN packages. Projections with prefix “02LiST2” are for PROVEN+POTENTIAL packages. The calculation sheets for BCR reported in the paper is also available to download at the same location.
